# Syria Profile of the Epidemiology and Management of Early Childhood Caries Before and During the Time of Crisis

**DOI:** 10.3389/fpubh.2019.00271

**Published:** 2019-09-24

**Authors:** Easter Joury

**Affiliations:** Centre for Dental Public Health and Primary Care, Institute of Dentistry, Queen Mary University of London, London, United Kingdom

**Keywords:** Syria, early childhood caries, oral health, pubic health interventions, humanitarian crisis

## Abstract

Syria has witnessed the greatest humanitarian crisis of forcibly displaced population since World War II. The present review aimed to outline Syria's profile of the epidemiology and management of early childhood caries (ECC). Before the crisis, the burden of ECC amongst Syrian pre-schoolers had been growing in prevalence and severity. Comparable data showed an increase in the burden of ECC amongst Syrian children aged five years, rising from 74% in 1991 to 81% in 2011, with a dmft value of 8.6. A similar increase was observed in the burden of ECC amongst Syrian children aged three years, rising from 50% in 1991 to 56% in 2011, with a dmft value of 6.1. Whilst there are no data on the burden of ECC during the current crisis, estimates could be extrapolated from data on the current burden of dental caries amongst Syrian primary school children living inside Syria or in informal settlements outside Syria. Such data suggested that the burden of ECC might have further increased amongst Syrian pre-schoolers during the crisis time. This is due to the crisis exacerbating effect on ECC risk factors, in terms of increasing the existing high sugar intake amongst Syrian pre-schoolers as well as increasing different barriers Syrian families face to fresh foods, sugar-free medicines, oral hygiene and fluoride products and accessing essential preventative dental care. Tackling the growing burden of ECC amongst Syrian pre-schoolers should not be postponed till post-crisis time. The seed work for relevant public health interventions could start and be embedded in different health and social initiatives taking place during the time of crisis. A number of public health interventions informed by relevant international and local (Syrian) studies conducted during the time of crisis have been suggested to tackle the burden of ECC amongst Syrian young children. They include a mix of upstream, midstream, and downstream interventions that aim to reduce sugar intake, improve feeding and oral hygiene practices, increase access to an appropriate source of fluoride and build the capacity of the Syrian dental and wider workforce to tackle the growing burden of ECC in Syrian pre-schoolers.

## Background

Syria is a Mediterranean country located in the Middle East, with a population of nearly 24 million people. Before the crisis, Syria was classified as a middle income country, with a health profile characterized by a high burden of non-communicable diseases (NCDs), including obesity, cardiovascular diseases, type 2 diabetes, and dental caries. Syria had a universal health care system with a vaccination coverage of nearly 99% (1).

Recently, Syria has witnessed the greatest humanitarian crisis of forcibly displaced population since World War II. According to the UNHCR, the recent marked increase in the global population of forcibly displaced people was driven mainly by the Syrian crisis (2). The latter has resulted in more than 13 million Syrians displaced within and outside Syria, of which over 5 million refugees are in neighboring countries: Lebanon, Jordan, Iraq, Turkey and Egypt (2). More than half of these refugees are children (2).

The present review aimed to outline Syria's profile in terms of the epidemiology and management of early childhood caries (ECC). More specifically, the objectives of the current work were to:

- Give an overview of the prevalence, trend, and impact of ECC before and during the Syrian crisis,
- Outline risk factors of ECC amongst Syrian pre-schoolers with a special focus on the role of the humanitarian crisis as an exacerbating factor, and
- Suggest evidence-based recommendations for the management of ECC in Syria from relevant international and local studies.

This review of literature searched MEDLINE via PubMed through August 2019. The following key words were used for the PubMed search: Syria^*^ and (oral or dent^*^). Additionally, other resources of gray literature relevant to oral health in Syria were searched. There was no language restriction.

## Prevalence, Trend, and Impact of ECC in Syria Before the Time of Crisis

Before the current crisis, Syria, like other countries in the Middle East, had a high burden of dental caries amongst children aged 5 years and under. Few studies have been conducted to estimate this burden. They varied in their methodological approaches and rigor. Most of them used the World Health Organization criteria for caries diagnosis and were conducted in Damascus (the capital city). Very few studies were carried out in other cities (e.g., Latakia and Aleppo). There are no Syrian national dental health survey data for pre-schoolers.

Taking into consideration the aforementioned limitations in terms of the quantity and quality of available (published and unpublished) studies, and using “comparable” data to make inference on ECC trends, it seems that the burden of ECC amongst Syrian 5-year-olds had been increasing from 74% in 1991 (3) to 81% in 2011 (4), with a dmft value of 8.6. The prevalence of severe ECC among pre-schoolers at the age of five was 77% (4). Amongst 3 year old Syrian children, a similar increase in the prevalence of ECC had been observed, rising from 50% in 1991 (3) to 56% in 2013 (4), with a dmft value of 6.1. The majority of pre-schoolers had untreated ECC (dt) (3, 4).

The impact of ECC on the quality of life of Syrian young children and their families was reported in a cross-sectional study (4). The latter used the Arabic version of Early Childhood Oral Health Impact Scale and included a sample of Syrian pre-schoolers with ECC aged between 3 and 5 years. The most frequently reported impact of ECC (experienced occasionally, often or very often) was pain (75%) followed by family feeling upset (52%) and having difficulty to chew food (48%).

## Prevalence, Trend, and Impact of Early Childhood Caries (ECC) in Syria During the Time of Crisis

During the Syrian crisis, no studies have to date been conducted to estimate the burden of ECC amongst pre-schoolers (including those who have or have not been internally or externally displaced). Emerging evidence from protracted Syrian refugee informal settlements in Lebanon suggests that the burden of ECC might have increased amongst refugee pre-schoolers. Such estimation is based on the findings related to the burden of dental caries amongst older Syrian children attending refugee primary schools in Bekkaa, Lebanon (5). The prevalence of dental caries has hit a record high of over 90% amongst these children. Oral pain was reported by 57%, of which 55% had moderate to severe oral pain and 38% had oral pain for more than a month (5). Furthermore, 9% had dental abscess. The difference in the number of teeth with untreated dental caries between children in a protracted displacement situation and those who have been displaced for <5 years was highly statistically significant (5). This suggested that protracted displacement might lead to an increase in the burden of dental caries amongst children and the deterioration of their oral health. Similar findings were reported by another study conducted on a small convenience sample of Syrian refugee children aged 6–12 years at al-Zaatari camp in Jordan (6).

Indeed, when comparing oral health between Syrian refugee children and their counterparts who had not been displaced or had been internally displaced within Syria it appears that the former had a much higher burden of dental caries compared with the latter. For example, a recent cross-sectional survey (7), conducted on Syrian children aged 8–12 years living in Damascus, reported 79.1% caries prevalence. The latter is more than 10% lower than the caries prevalence found amongst Syrian refugee children of the same age group (5). It is worth mentioning that Damascus is one of the Syrian cities that have had the highest prevalence of dental caries and now is hosting a mix of children that had not been displaced as well as children who had been internally displaced in Syria. This implies that the prevalence of oral diseases amongst Syrian refugee children in low and middle-income countries is much higher than the highest prevalence of oral diseases found in the country of origin (Syria). In other words, the Syrian crisis affected negatively the whole Syrian child population on a scale of intensity that is proportionate to the extent of displacement. Syrian children who have not been displaced are less affected compared with their internally displaced counterparts, who in turn are less affected than their refugee or externally displaced peers.

Syrian refugee caregivers had voiced strong concerns about their children's oral health in humanitarian settings and its considerable impact on families (8). Child's dental pain had been repeatedly reported to cause considerable distress for Syrian refugee parents (and children) and served as a trigger for deeper feelings of anger, frustration and helplessness (8). This might explain why international humanitarian organizations such as the UNICEF and UNHCR have become highly interested in addressing the burden of dental caries amongst displaced and refugee children (9, 10).

## Risk Factors for ECC Amongst Syrian Children

A number of studies explored the proximal (behavioral) and distal (social) risk factors of ECC amongst Syrian pre-schoolers. Behavioral risk factors focused on poor feeding practices, high sugar intake, poor oral hygiene, limited exposure to an appropriate source of fluoride and low dental care utilization. The role of the current Syrian humanitarian crisis as an exacerbator for ECC risk factors was noted in the literature.

### Poor Feeding Habits Amongst Syrian Pre-schoolers

Although breastfeeding is the most common type of infant feeding in Syria some poor feeding practices were identified. A recent cross-sectional study that targeted 1 year old Syrian infants attending vaccination clinics in Damascus found that 47% of infants were breastfed, 42% were breast and bottle fed and 11% were exclusively bottle fed (11). The use of exclusive bottle feeding was more common amongst working mothers (11). Nearly 50% of Syrian infants who used the bottle were put to sleep with the bottle (11, 12), increasing therefore the risk of having ECC. Additionally, other poor infant's feeding practices such as the prolonged use of bottle-feeding (beyond the child's first birthday) were identified among Syrian pre-schoolers with ECC (12).

### High Sugar Intake Amongst Syrian Pre-schoolers

A number of studies have highlighted the high intake of sugar (i.e., free sugars) among Syrian pre-schoolers, which tends to start early during their first year of life. Nearly 43% of Syrian infants had high frequency of sugar intake (>4 times a day) (11). Furthermore, 75% had sugar added to their beverages (11). Amongst Syrian pre-schoolers aged 3 to 5 years, 86% had 3–4 meals and/or snacks with high sugar content daily (12).

In regard to child's liquid medicines available on the Syrian market, the vast majority has sugar. The number of sugar-free medicines has dropped during the current Syrian crisis (13). Additionally, a cross-sectional study found that only 18% of Syrian pediatricians were aware of sugar-free liquid medications available on the Syrian market and only 10% prescribed such medications (13).

### Poor Oral Hygiene and Limited Access to an Appropriate Source of Fluoride Amongst Syrian Pre-schoolers

Less than six percent of 1 year old infants had their teeth cleaned regularly with a toothbrush or piece of clean gauze (11). The percentage of pre-schoolers aged 3–5 years who had their teeth cleaned at least twice a day had increased from 1.4% in 2007 (12) to 12.3% in 2011 (4). Fluoride toothpaste was rarely used amongst Syrian infants (11).

Community-based water or salt fluoridation schemes have to date not been possible in Syria (3). Very few Syrian areas contain naturally fluoridated water at either sufficient levels to protect tooth decay (nearly 1 ppm) or excessive levels that might cause fluorosis (e.g., Palmyra and Alhasak) (3).

A recent study highlighted that preventive oral care products, including fluoride toothpastes, gels, and varnishes, that adhered to evidence-based recommendations were either few in number or not available on the Syrian market (14). Several fluoride toothpastes had fluoride concentrations that were low (i.e., below the effective concentration for caries prevention), or high with potential long-term toxic effects in children. Thirteen toothpastes with fluoride concentration <1,000 ppm claimed to have anticaries effects. Another three fluoride toothpastes did not declare their fluoride concentration. Additionally, 17 toothpastes with high fluoride concentration were available for purchase without prescription and had instructions for use in children younger than the safe evidence-based recommended age. In terms of fluoride gels and varnishes, only three fluoride gels were available (14). No fluoride varnish was found (14), which is known to be more effective than the gels for prevention of dental caries (15). During the current Syrian crisis, the number of preventive oral care products available on the market has decreased and some of the currently available products are still non-adherent to evidence-based recommendations (14).

### Low Dental Care Utilization Amongst Syrian Pre-schoolers

Low dental care utilization is another risk factor of ECC amongst Syrian pre-schoolers. Less than two percent of young Syrian children had visited the dentist by their first birthday (11).

### Social Risk Factors of ECC Amongst Syrian Pre-schoolers

A few studies documented social inequalities in ECC amongst Syrian pre-schoolers (4). Socioeconomic inequalities were also identified in the frequency of sugar intake among Syrian infants (11). Infants whose fathers were not working were more likely to have high frequency of sugar intake compared to their counterparts whose fathers were working. Furthermore, Syrian infants whose mothers had low knowledge about infant's oral health behavior or high frequency of sugar intake were more likely to have high frequency of sugar intake (11). Mother's knowledge of infant's oral health behavior and her frequency of sugar intake explained the socioeconomic inequality in infant's frequency of sugar intake. This suggested the importance of addressing such factors (i.e., mother's knowledge of infant's oral health behavior and her frequency of sugar intake) to reduce inequalities in pre-schoolers' oral health. The vast majority of Syrian mothers reported the importance of primary teeth (94%) and voiced their desire to receive information about infant's oral health (99%) (11). Only 6% of Syrian mothers reported receiving information about child's oral health (11).

### The Syrian Humanitarian Crisis as an Exacerbator for ECC Risk Factors Amongst Syrian Pre-schoolers

The Syrian humanitarian crisis has created many political, economic and social negative circumstances that had a profound impact on different living aspects of the Syrian population. The levels of mal- and under-nutrition had increased as a result of the disturbance in food channels during crisis time. For example, many Syrian refugee families mentioned barriers related to the availability and affordability of fresh foods (e.g., fresh fruits and vegetables) (8). The latter is expected in view of complex emergences and disaster context (16). Foods and drinks with high sugar content are usually more available at the camps' tuck shops at affordable prices. Additionally, due to forced displacement the family's daily routine is likely to be negatively affected and parents and carers are more likely to experience loss of control over their children's behaviors. The latter might be one of the reasons behind the increase in children's sugar consumption.

Similar to fresh food, refugee parents and carers face multiple barriers to oral hygiene products and accessing dental care, leading to the deterioration of their children's oral health.

## Recommendations for the Management of ECC in Syria

Tackling the growing burden of ECC amongst Syrian pre-schoolers should not be postponed till post-crisis period. It is acknowledged that there are many barriers to implement public health interventions to tackle the ECC burden in Syria during crisis time as opposed to post-crisis period. Nonetheless, the seed work for such interventions could start and be embedded in different health and social initiatives taking place at the time of crisis.

The following suggestions for public health interventions are not only guided by evidence emerging from international studies, but also and more importantly they are informed by evidence emerging from local studies showing the feasibility, acceptability and preliminary effectiveness and cost-effectiveness of such interventions within the Syrian context during crisis time. Yet, it should be acknowledged that institutionalizing such recommendations and rolling them out across the country during the time of crisis would need further larger national studies dedicated for investigating barriers and enablers to implementing these recommendations on a national scale. The dependency on external aids should be minimized, as such aids are unlikely to create sustainable changes (17).

Syrian oral health strategies should aim to decrease sugar intake, promote good feeding, and oral hygiene practices, increase exposure to an appropriate source of fluoride and build the capacity of the Syrian dental and wider workforce to promote oral health and tackle the growing burden of ECC across multi-sectoral settings.

A mix of sustainable and affordable upstream, midstream, and downstream interventions are recommended. The principles of propionate universalism (18) should guide the roll out of suggested interventions to ensure that the scale of their delivery is proportionate to the intensity and levels of needs.

### Reducing Sugar Intake Amongst Syrian Pre-schoolers

Reducing sugar intake would not only improve the oral health of Syrian pre-schoolers, but also it would improve their general health. High sugar intake is a common risk factor for child's mal-/under-nutrition and dental caries.

The main effective and cost-effective means to reduce sugar intake amongst Syrian pre-schoolers is to develop and implement public health policies with related regulations and legislations. The following is evidence-based recommendations to inform governmental leaders and policymakers regarding potential public health policies and strategies to reduce sugar consumption particularly amongst young Syrian children.

The Syrian government should formulate country-specific goals to decrease the high levels of sugar consumption and reach the latest recommended maximum of 5% of total energy (19). This equates to ~22 g/day for 4–6 year olds and 16 g/day for 1–3-year-old children (20).

Furthermore, the Syrian government should adopt the Health in All Policies (HiAP) framework. The latter would aid in developing intersectoral partnership among the Syrian ministries and yield large returns on investment as a result of targeting a common risk factor (i.e., high sugar intake), and preventing thereby a spectrum of childhood diseases (e.g., dental caries, malnutrition and obesity) (21, 22) as well as other sugar-related NCDs that young children are likely to develop over the life course (e.g., adulthood obesity, type 2 diabetes and dental caries) (23, 24). The HiAP approach should include:

- Introducing taxes on imported or locally manufactured sugary foods and drinks in Syria: sugar is easy to tax because it goes through a long chain. Introducing a tax on sugar as a mass commodity is the simplest form of sugar taxation (25). It is recommended to have an increase by at least 20% in the retail price of sugary items to achieve a desirable effect on consumers' demand and hence consumption (25). No taxes should be imposed on sugar-free foods, drinks or medicine products. Sugar-tax returns could be used to subsidize the prices of fresh vegetables, fruits, and sugar-free medicines available on the Syrian market.
- Introducing regulations and legislations pertaining to the Syrian food industry: the Syrian government should put pressure on the food and pharmaceutical industry to reduce the sugar content in their products and offer a wide range of sugar-free alternatives (25). Appropriate legislations are needed to support such efforts. Additionally, clear and unbiased labeling is key. Products containing more than 2.5% free sugars should be labeled as “high.”
- Policies on food provided particularly in nurseries, kindergartens, maternal, and child community centers in Syria: packed lunches and food provided in nurseries and other young children's settings should be sugar free or at least sugar reduced (25). Fruit juices and sugar-containing treats and confectionaries should be limited to a maximum of 2.5% energy intake.
- Restrictions on the advertisement of sugary foods and drinks in Syria: the Syrian government should set more stringent codes of practice and restrictions on the advertisement of sugary foods and drinks, particularly to children (26).
- Calling the Regional Office of the World Health Organization for the Eastern Mediterranean to review its recommendation on adding little quantities of sugary items to baby's food (27). The latter is still communicated to Syrian mothers in early childhood programmes.
- All the aforementioned policies should be supported with initiatives to increase the public awareness of the need to reduce the intake of sugary food and drinks from infancy throughout the life course, using integrated pre/post-natal interventions within the Syrian national nutrition and maternal and child programmes (e.g., the Syrian breastfeeding and vaccination programmes). These whole population interventions should be supported by other tailored oral health education and dietary interventions to target vulnerable or disadvantaged Syrian families to reduce social inequalities (11).

### Promoting Good Feeding and Oral Hygiene Practices and Increasing Exposure to an Appropriate Source of Fluoride Amongst Syrian Pre-schoolers

Public health interventions should aim to promote good feeding and oral hygiene practices as well as increase exposure to an appropriate source of fluoride amongst Syrian pre-schoolers. Fluoride toothpaste might be the only vehicle that could provide Syrian infants and pre-schoolers with access to fluoride (28). However, this should not cease efforts to explore the feasibility of salt or water fluoridation schemes in Syria. Water fluoridation mapping and feasibility studies were conducted in Syria during the 1980s and thus they are quite dated now. The following is evidence-informed recommendations based on a number of recent local studies (14, 28–30):

- Running community-based programmes to establish tooth-brushing with fluoride toothpaste and bottle feeding termination practices amongst Syrian infants: a randomized controlled trail demonstrated the feasibility, acceptability and preliminary effectiveness and cost-effectiveness of such interventions in the Syrian context (28). This trail integrated the intervention within the Syrian national vaccination programme and delivered an oral health promotion package free of charge, without health workers' counseling. The package included an infant oral health pamphlet, a baby toothbrush, fluoride toothpaste (1,000 ppm) and a trainer cup. Infants who received this package were less likely to have old plaque (as a measure of tooth brushing behavior) and more likely to stop bottle-feeding than their counterparts who did not receive the package.
- Carrying out national water and salt fluoridation mapping and situation analyses: this would assess the feasibility and acceptability of rolling out water or salt fluoridation schemes. Such schemes have shown in other countries effectiveness not only in decreasing ECC but also in reducing oral health inequalities amongst pre-schoolers.
- Running targeted fluoride varnish or silver diamine fluoride school-based programmes for vulnerable/disadvantaged groups of pre-schoolers, such as those with special needs (29, 30).
- The Syrian Ministry of Health should review its regulations and carry out necessary reforms for fluoride products so that they become subject to drug monitoring systems to ensure effective and safe fluoride concentrations in such products (14).
- Effective communication should be established with Syrian manufacturers to reformulate their preventive oral care products to have evidence-based effective and safe fluoride concentrations, labeling and instructions for use (14). The return on investment of producing silver diamine fluoride and fluoride varnish locally should be investigated. Additionally, strict regulations should be imposed on imported preventive oral care products. All available self-care products should have instructions for use written in Arabic, so they can be read and understood by Syrian consumers (14).
- The Syrian Arab Committee for Measurements and Standards needs to update its standards related to preventive oral care products (14). The latest update took place in 1995 and it is quite dated now. Several preventive care products that are available on the Syrian market include instructions for use that contradict current evidence-based international recommendations (14).

### Building the Capacity of the Syrian Dental and Wider Workforce to Tackle the Burden of ECC Amongst Syrian Pre-schoolers

As there is “No health without health workforce” (31), it is important to ensure that the Syrian dental workforce has the right capacity and capability to tackle the growing burden of ECC. Before the crisis, there were nearly 20,000 registered Syrian dentists with nearly 2,000 new dental graduates annually. Although there are no data on the current capacity and capability of the Syrian dental workforce it is expected that these two aspects have been negatively affected during the current Syrian crisis. A brain drain of the Syrian dental workforce had taken place during the crisis time. Thus, it is important to develop capacity building strategies and interventions to ensure that the Syrian dental and wider workforce is able to address the burden of ECC in Syria. The following is recommendations based on a number of recent regional and local studies (14, 17, 29, 30):

- Reforming and modernizing healthcare curricula to ensure the competencies around oral health promotion and ECC evidence-based prevention are embedded across dental, medical, and pharmacy curricula taught in Syrian universities. The modernization of the dental public health discipline in Damascus University, with its outreach programme and activities “Syrian Smiles” (28, 29) is a successful example of building the competency of dental under- and post-graduates in oral health promotion and prevention for children during the time of the Syrian crisis.
- Promoting skill mix in dentistry in Syria through introducing appropriate governance and educational reforms: such reforms would ensure the presence of (i) a clear scope of practice for Syrian dental nurses and hygienists; (ii) regulations and legislations governing the work of dental care professionals (DCP) within the Syrian healthcare system and dental services; and (iii) modernized DCPs curricula that include training on up-to-date evidence-based preventive care for children. The possibility of training community health care workers (CHCWs) on delivering outreach basic preventative care to families with young children could be considered. Yet, supportive evidence is needed regarding its feasibility, acceptability and cost-effectiveness.
- Running continuing professional development (CPD) courses for the Syrian dental and wider workforce (e.g., doctors, pediatricians, midwives, and pharmacists etc.) dedicated to promoting the oral health of pre-schoolers.
- The Syrian General Dental Association should distribute a user-friendly booklet for preventive care to assist dental practitioners in implementing evidence-informed preventive care (14).
- Establishing a national team responsible of conducting regular national oral health surveys for Syrian children aged 5 years: this is highly important to monitor the trends in ECC among Syrian children and evaluate the national and local oral health interventions aiming to tackle the ECC burden in Syria.

## Conclusion

Syria has witnessed the greatest humanitarian crisis of forcibly displaced population since World War II. The burden of ECC amongst Syrian pre-schoolers had continued to grow in prevalence and severity. The humanitarian crisis had exacerbated ECC risk factors, in terms of increasing the existing high sugar intake amongst Syrian pre-schoolers as well as increasing different barriers Syrian families face to fresh foods, sugar-free medicines, oral hygiene and fluoride products and accessing essential preventative dental care. The seed work to tackle the burden of ECC could start and be embedded in different health and social initiatives taking place during the time of crisis. A number of public health interventions informed by international and local (Syrian) studies have been suggested to tackle the ECC burden amongst Syrian young children. They include a mix of upstream, midstream, and downstream interventions that aim to reduce sugar intake, improve feeding and oral hygiene practices, increase access to an appropriate source of fluoride and build the capacity of the Syrian dental and wider workforce to tackle the growing burden of ECC in Syrian pre-schoolers.

## Author Contributions

The author confirms being the sole contributor of this work and has approved it for publication.

### Conflict of Interest

The author declares that the research was conducted in the absence of any commercial or financial relationships that could be construed as a potential conflict of interest.
